# Low-value pharmaceutical care among Dutch GPs: a retrospective cohort study

**DOI:** 10.3399/BJGP.2021.0625

**Published:** 2022-03-22

**Authors:** Joris LJM Müskens, Simone A van Dulmen, Tjerk Wiersma, Jako S Burgers, Karin Hek, Gert P Westert, Rudolf B Kool

**Affiliations:** Radboud Institute for Health Sciences, IQ healthcare, Radboud University Medical Centre, Nijmegen.; Radboud Institute for Health Sciences, IQ healthcare, Radboud University Medical Centre, Nijmegen.; Dutch College of General Practitioners, Utrecht.; Department of General Practice, School CAPHRI, Maastricht University, Maastricht; senior consultant, Dutch College of General Practitioners, Utrecht.; Nivel, Netherlands Institute for Health Services Research, Utrecht.; *‘Doen of laten?’*, IQ Healthcare, Radboud Institute for Health Sciences, IQ Healthcare, Radboud University Medical Centre, Nijmegen.; *‘Doen of laten?’*, IQ Healthcare, Radboud Institute for Health Sciences, IQ Healthcare, Radboud University Medical Centre, Nijmegen.

**Keywords:** general practice, general practitioners, inappropriate prescribing, medical overuse

## Abstract

**Background:**

Low-value pharmaceutical care exists in general practice. However, the extent among Dutch GPs remains unknown.

**Aim:**

To assess the prevalence of low-value pharmaceutical care among Dutch GPs.

**Design and setting:**

Retrospective cohort study using data from patient records.

**Method:**

The prevalence of three types of pharmaceutical care prescribed by GPs between 2016 and 2019 were examined: topical antibiotics for conjunctivitis, benzodiazepines for non-specific lower back pain, and chronic acid-reducing medication (ARM) prescriptions. Multilevel logistic regression analysis was performed to assess prescribing variation and the influence of patient characteristics on receiving a low-value prescription.

**Results:**

Large variation in prevalence as well as practice variation was observed among the types of low-value pharmaceutical GP care examined. Between 53% and 61% of patients received an inappropriate antibiotics prescription for conjunctivitis, around 3% of patients with lower back pain received an inappropriate benzodiazepine prescription, and 88% received an inappropriate chronic ARM prescription during the years examined. The odds of receiving an inappropriate antibiotic or benzodiazepine prescription increased with age (*P*<0.001), but decreased for chronic inappropriate ARM prescriptions (*P*<0.001). Sex affected only the odds of receiving a non-indicated chronic ARM, with males being at higher risk (*P*<0.001). The odds of receiving an inappropriate ARM increased with increasing neighbourhood socioeconomic status (*P*<0.05). Increasing practice size decreased the odds of inappropriate antibiotic and benzodiazepine prescriptions (*P*<0.001).

**Conclusion:**

The results show that the prevalence of low-value pharmaceutical GP care varies among these three clinical problems. Significant variation in inappropriate prescribing exists between different types of pharmaceutical care — and GP practices.

## INTRODUCTION

Low-value care, defined as care that is unlikely to benefit the patient given the potential harm, cost, available alternatives, and patient preferences, is considered one of the most complex problems in modern health care.^1^^,^^2^ In an effort to support clinicians in their daily practice, professional bodies, such as the Dutch College of General Practitioners, have published >120 evidence-based clinical practice guidelines.^3^ However, despite the wide distribution and promotion of these guidelines, studies show that adherence among Dutch GPs could be improved.^4^^–^10 Up to one-third of Dutch GP pharmaceutical prescriptions could be of low value.^6^ International studies show that low-value GP prescribing is also common outside of the Netherlands.^2^^,^^11^^–^17

Obtaining insight into the prevalence of low-value prescribing is an essential first step in improving practice.^18^ Although some assessments of low-value prescribing among Dutch GPs exist, these are outdated and conducted using data that are not nationally representative.^4^^,^^19^^–^21 The aim of the current study was therefore to quantify the prevalence and variation in low-value pharmaceutical treatments among GPs using data from national medical records.

Three recommendations were selected from the set of GP guidelines that clearly emphasise that physicians should refrain from prescribing medication except when specific indications are met:

- the prescription of local antibiotics for an infectious conjunctivitis;^22^
- the prescription of benzodiazepines in the treatment of non-specific lower back pain; and^23^
- the chronic prescription or continuation of acid-reducing medication (ARM).^24^

Box 1 contains a detailed description of the rationale behind the selection (and Supplementary Box S1 provides a description of the operationalisation of each recommendation).

**Box 1.** Rationale behind the recommendations selected

*The prescription of local antibiotics for an infectious conjunctivitis.*^22^ A Dutch study from 2007 showed that up to 80% of conjunctivitis episodes were inappropriately treated with a topical antibiotic.^20^ The indications for prescribing an antibiotic for conjunctivitis have not changed since then. One study from the US showed that the number of conjunctivitis diagnoses has increased between 2005 and 2014, and the percentage of low-value antibiotic prescriptions slightly decreased from 18.3% to 17.2%.^25^ It would be interesting to see whether the prevalence of antibiotic prescription in the Netherlands has also changed over time.
*The prescription of benzodiazepines in the treatment of non-specific lower back pain*.^23^ Lower back pain is one of the most prevalent conditions seen in general practice.^26^ Its treatment, however, is complex.^27^ Studies indicate that both inappropriate imaging^11^^,^^12^^,^^28^^,^^29^ and prescribing of opioids^27^^,^^30^^,^^31^ are highly prevalent. However, there is a lack of information about the prevalence of inappropriate prescribing for lower back pain in the Netherlands.
*The chronic prescription or continuation of acid-reducing medication (ARM) without indication.*^24^ Inappropriate prescription of ARM, predominantly proton pump inhibitors, has been shown to be an international problem.^32^^–^37 However, the extent of this problem in the Netherlands is unknown.
Supplementary Box S1 and Supplementary Information S1 contain a detailed description of how each recommendation was operationalised and the definitions used.

Through quantification of these prescribing practices, a clearer view of low-value prescribing among GPs in the Netherlands should be obtained. This is a first step in addressing the specific issue of low-value GP prescribing. In addition to studying both the prevalence and the variation in prescribing behaviour, another aim was to identify the characteristics of patients associated with low-value prescribing.

**Table:** How this fits in

The extent of low-value prescribing among Dutch GPs is largely unknown. Obtaining insight into the prevalence of low-value care is an essential first step in the process of improving the quality of care. In close collaboration with the Dutch College of General Practitioners, this study assessed three types of low-value GP pharmaceutical care relating to conjunctivitis, lower back pain, and acid-reducing medication. This information could be used to design a campaign, nationally or locally, to improve guideline-consistent prescribing behaviour.

## METHOD

### Design and database

A retrospective cohort study with data derived from the Nivel Primary Care Database (Nivel-PCD) was conducted. The Nivel-PCD contains care data routinely collected from electronic medical records from GPs throughout the Netherlands. The data were obtained from 529 GP practices, representing approximately 2 million registered patients.^38^ The sample was shown to be representative for the total population of Dutch primary care practices.^39^^–^41 The database contains longitudinal information regarding patient characteristics such as age, sex, GP consultations, diagnoses, and drug prescriptions. Diagnoses are recorded using the International Classification of Primary Care version 1 (ICPC-1). Prescriptions are recorded using the Anatomical Therapeutic Chemical classification system (ATC). This study was approved by the relevant governance bodies of the Nivel-PCD (nr. NZR00320.001).

### Cohort selection

For each of the three low-value care issues examined, patients with relevant episodes were extracted from the Nivel-PCD. Next, the prescription files for each type of pharmaceutical care were filtered for prescriptions associated with relevant ICPC codes. The resulting selection was then used in the analysis. Supplementary Box S1 contains an overview of the ATC and ICPC codes used to define the patient population for each of the recommendations. The analysis only included GP practices where sufficient prescription data of high quality were available between 2016 and 2019. GP practices had to meet the following criteria to be included in the analysis:

- at least 85% of the prescriptions were encoded with a valid ATC code;
- a minimum of 46 weeks of prescription data had to be present; and
- a minimum of 500 patients per practice should be included in the data.

### Data analyses

The assessments were performed using a patient-indication lens, as described by Chalmers *et al*,^42^ meaning that patients were only included with a specific indication in the selected denominators. The primary outcome is the percentages of patients with an indication who received a low-value prescription at least once. Analysis was performed using Stata (version 16). Data visualisation was carried out using R (version 3.6.3) and the R-package ggplot2.

### Practice variation

Variation among GP practices was assessed through multilevel logistic regression analysis over 2019, with random effects at the practice level. Before performance of multilevel logistic regression, variance inflation factors (VIF) were calculated to test for collinearity among the included variables. In order to prevent the standard errors of the (multilevel) regression coefficients becoming too large, GP practices with <5 cases of low-value prescribing, or <30 patients with a relevant indication for the type of care examined, were excluded from the analysis.

Intraclass correlation coefficients (ICC) were calculated to assess variation in low-value prescribing between GP practices.^43^ C-statistics were calculated for models with, and without, a random effect for the level of the practice. The presence of higher c-statistics associated with the models with a random intercept for the level of practice suggest that these models have more predictive accuracy compared with the models without the random intercept. The difference between both C-statistics was used as a measure for variation among GP practices.

Additionally, whether or not a significant correlation exists between the prescription rates of the three types of low-value pharmaceutical care over 2019 was assessed. Correlations were assessed using the Pearson correlation coefficient for normally distributed variables and the Spearman correlation coefficient for non-normally distributed variables. Normality was assessed using both density plots and the Shapiro–Wilk test.

### Case-mix variables

The following patient variables were included in the models to assess their influence on the odds of receiving low-value care: age, sex, and neighbourhood socioeconomic status (SES). These case-mix variables were selected based on previous research indicating that these factors affect the amount of care patients require, receive, and have access to.^44^^–^48 SES scores from 2017 were derived from the Dutch Institute for Social Research.^49^ Patients were assigned to one of five categories (lowest, below average, average, above average, or highest) based on quintiles. In addition to these patient characteristics, the number of patients registered at each GP practice was also included in the analysis and categorised as a small, medium, or large practice, based on the division of each population into tertiles.

## RESULTS

**Figure 1.**
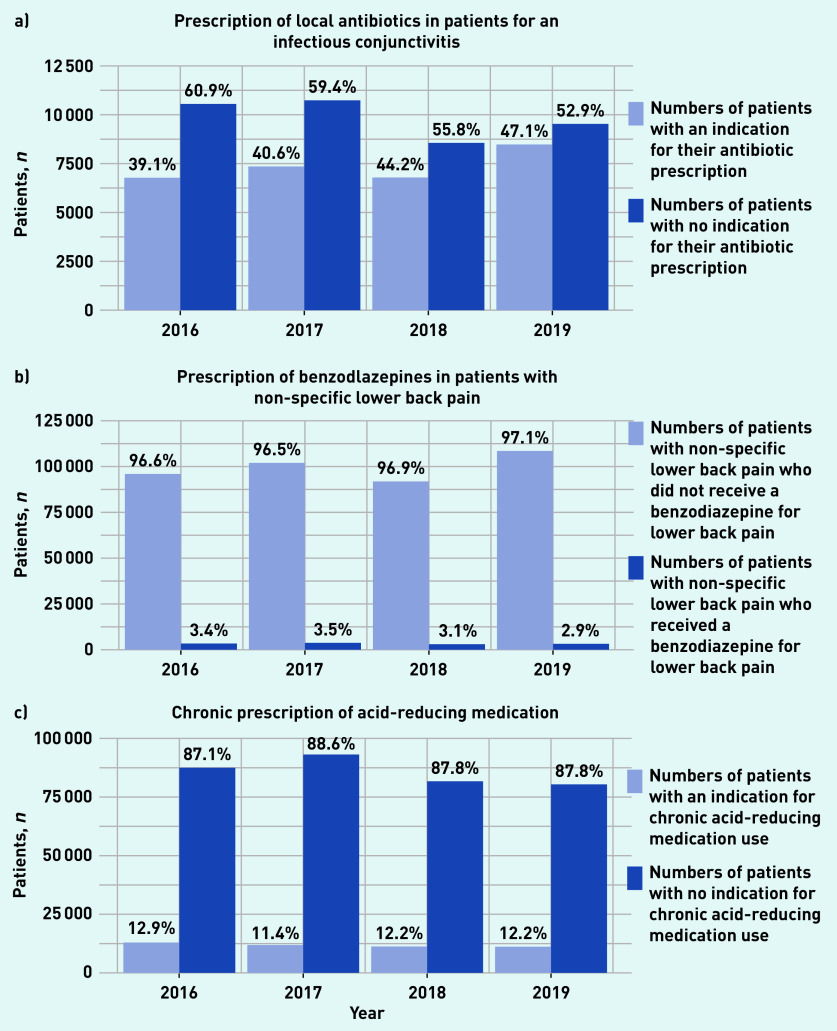
*Estimates of the prevalence of patients receiving one of three types of low-value care. Associated with a) conjunctivitis; b) non-specific lower back pain; and c) acid-reducing medication.*

Table 1 and Figure 1 provide a summary of the study results. Patients with an episode of infectious conjunctivitis were regularly prescribed local antibiotics without appropriate indication. The proportion of patients inappropriately prescribed antibiotics decreased from 61% to 53% between 2016 and 2019. The chronic use of ARMs without an appropriate indication was highly prevalent. Between 2016 and 2019, around 88% of patients with a chronic ARM prescription lacked an appropriate indication. The prescription of benzodiazepines for lower back pain remained around 3% over the 4 years. More detail can be found in Supplementary Table S1.

**Table 1.** Overview of assessment outcomes

**Recommendation**	**2016**	**2017**	**2018**	**2019**
**1. Do not prescribe a local antibiotic for an infectious conjunctivitis due to a banal pathogen, unless for a high-risk patient**				
Practices included, *n*	316	329	296	346
Patients with (at least) one episode of conjunctivitis (denominator), *n*	17 332	18 076	15 345	17 994
Patients who received appropriate treatment, *n* (%)	6778 (39.1)	7339 (40.6)	6789 (44.2)	8472 (47.1)
Patients with no clear indication for their antibiotic prescription (numerator), *n* (%)	10 554 (60.9)	10 737 (59.4)	8556 (55.8)	9522 (52.9)

**2. Do not prescribe benzodiazepines in patients with non-specific lower back pain**				
Practices included, *n*	313	328	296	346
Patients with (at least) one episode of lower back pain (denominator), *n*	99 262	105 641	94 685	111 703
Patients who did not receive a benzodiazepine or received one for a different indication, *n* (%)	95 909 (96.6)	101 922 (96.5)	91 742 (96.9)	108 441 (97.1)
Patients with no clear indication for benzodiazepine use (numerator), *n* (%)	3353 (3.4)	3719 (3.5)	2943 (3.1)	3262 (2.9)

**3. Do not chronically prescribe or continue ARM, without proper indication**				
Practices included, *n*	284	276	250	245
Patients with chronic prescription of ARM (denominator), *n*	100 319	105 043	93 053	91 563
Patients with an indication for chronic ARM use, *n* (%)	12 931 (12.9)	11 941 (11.4)	11 334 (12.2)	11 174 (12.2)
Patients with no clear indication for chronic ARM use (numerator), *n* (%)	87 388 (87.1)	93 102 (88.6)	81 719 (87.8)	80 389 (87.8)

*ARM = acid-reducing medication.*

### Variation at the practice level

**Figure 2.**
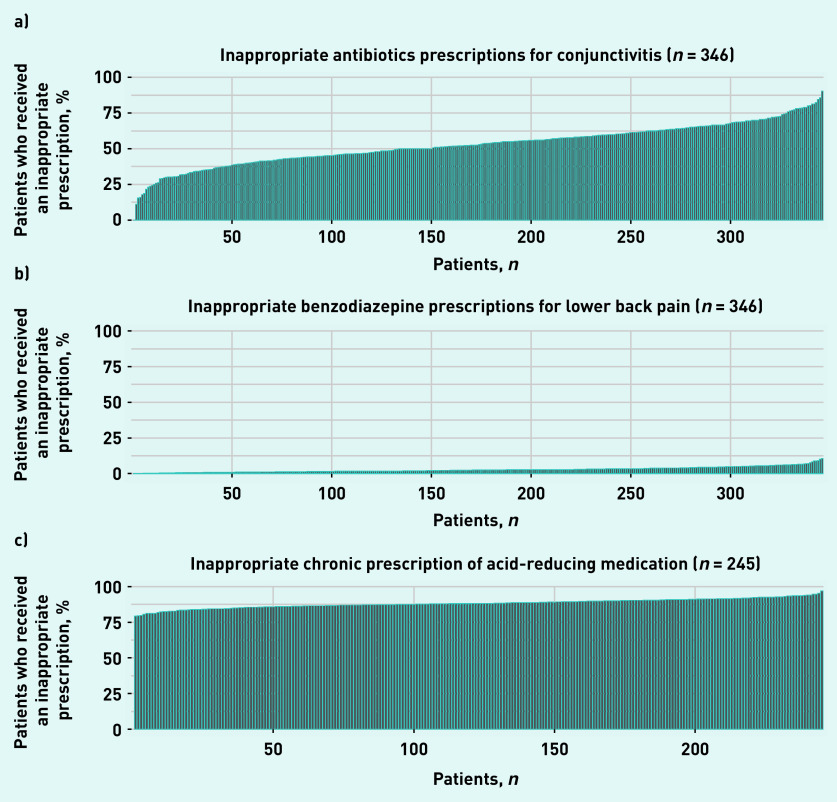
*Proportion of patients in each practice who received each of the low-value prescriptions for care at least once during 2019. Associated with a) conjunctivitis; b) lower back pain; and c) acid-reducing medication. (The practice numbers do not directly correlate to the practice numbers as provided in Supplementary Table S3.)*

Figure 2 shows the prevalence of low-value prescribing among GP practices during 2019. A large variation in the proportion of patients receiving at least one non-indicated prescription for antibiotics for conjunctivitis (Figure 2a) was observed. This varied between 0% and 90.3% (median 52.8%). Benzodiazepines were prescribed largely in line with the guidelines, showing limited variation. Between 0% and 11% (median 3.0%) of the patients with lower back pain at each of the included GP practices received an inappropriate prescription. ARMs were prescribed chronically without an appropriate indication in between 79% and 97% of the GP practices included (median 88%).

Comparison of the rates of non-indicated prescription of these three low-value pharmaceutical GP care types across practices only revealed a significant weak positive correlation (correlation coefficient 0.17) between the rate of low-value antibiotic and low-value benzodiazepine prescriptions (see Supplementary Table S2). No other significant correlations were identified.

Supplementary Table S3 contains an overview of the rates of low-value care for each of the examined types of care across practices in 2019. After adjusting for case-mix variables, the ICCs at the practice level for each of the prescriptions for low-value care ranged from 6% to 10% (Table 2). Analysis of the VIF factors revealed that little or no collinearity exists among the variables included in the analysis (see Supplementary Table S4). The C-statistics of the models with a random effect at the practice level were significantly higher for all three types of low-value GP care examined, compared with the models without random effect. Supplementary Table S5 presents an overview of the contribution of each case-mix variable to the final model.

**Table 2.** Overview of model characteristics

**Recommendation**	**ICC practice (95% CI)**	**C-statistic model with random-effect practice (95% CI)**	**C-statistic model without random-effect practice (95% CI)**
1. Do not prescribe a local antibiotic for an infectious conjunctivitis due to a banal pathogen, unless for a high-risk patient	0.08 (0.06 to 0.11)	0.65 (0.64 to 0.66)	0.54 (0.53 to 0.55)
2. Do not prescribe benzodiazepines in patients with non-specific lower back pain	0.10 (0.07 to 0.15)	0.67 (0.66 to 0.68)	0.59 (0.58 to 0.60)
3. Do not chronically prescribe or continue acid-reducing medication, without proper indication	0.06 (0.03 to 0.09)	0.72 (0.71 to 0.72)	0.70 (0.69 to 0.70)

*ICC = intraclass correlation coefficient.*

### Patient characteristics associated with receiving low-value prescriptions

The inappropriate prescription of antibiotics for conjunctivitis and benzodiazepine for non-specific lower back pain showed a significant increase in odds with increasing age (*P*<0.001, Supplementary Table S5). Conversely, patients were less likely to receive an inappropriate chronic prescription of ARMs with increasing age (*P*<0.001). Sex (*P*<0.001 and SES (*P*<0.05) significantly affected the odds of receiving a non-indicated chronic ARM prescription. Females were slightly less prone to receiving a non-indicated chronic ARM prescription.

Patients showed significantly increased odds of receiving an inappropriate ARM with increasing SES. Furthermore, SES resulted in only a small significant increase in odds of receiving an inappropriate antibiotic for conjunctivitis, when comparing patients with the average SES with the lowest category (*P*<0.05).

**Figure 3.**
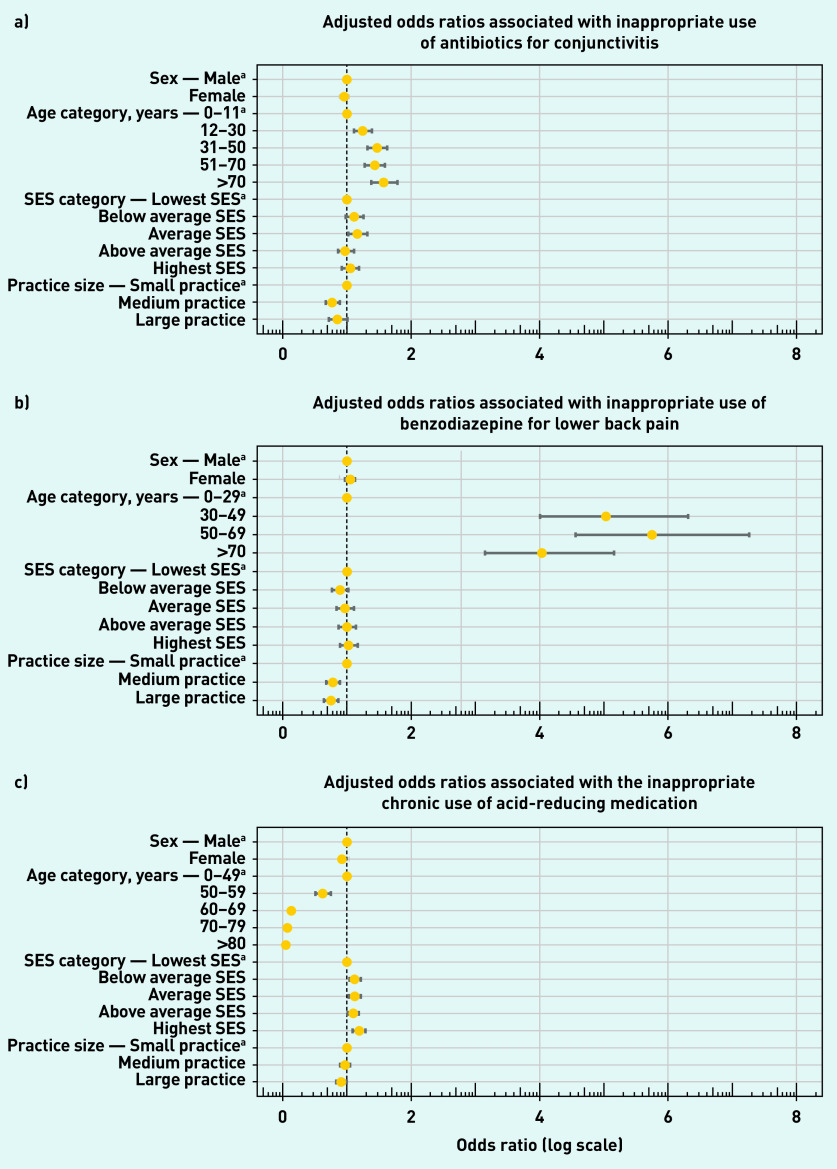
***Adjusted odds ratios and confidence intervals associated with patient characteristics for all three types of low-value GP care. Associated with a) conjunctivitis; b) lower back pain; and c) acid-reducing medication.*** *^a^****Reference categories. SES = socioeconomic status.***

The size of the GP practice significantly affected the odds of receiving any of the three types of low-value care examined. In general, larger GP practices were less prone to providing any of the three types of low-value care compared with smaller practices (*P*<0.05). In the case of chronic ARM use only, however, it appeared that medium-sized practices did not significantly differ in odds from the smaller ones (Figure 3).

## DISCUSSION

### Summary

This study showed that the prescription of low-value pharmaceutical GP care varied depending on the clinical problem. Inappropriate prescriptions of both antibiotics for conjunctivitis and ARMs were highly prevalent but the proportion of patients with lower back pain receiving a benzodiazepine was small. Large variation in pharmaceutical treatment was found for the prescription of a non-indicated antibiotic for conjunctivitis, whereas limited variation was found in the inappropriate prescription of benzodiazepines or non-indicated chronic ARM prescriptions. The analysis of correlation among the practices over 2019 only revealed a significant weak positive correlation between the rate of low-value antibiotic and benzodiazepine prescriptions.

The odds of a patient receiving any of the three low-value treatments was significantly affected by age. Males were found to have significantly higher odds of receiving a non-indicated chronic ARM prescription compared with females, and the odds of patients receiving an inappropriate ARM significantly increased with increasing neighbourhood SES. Apart from ARMs, SES only showed a small significant increase in the odds of receiving an inappropriate antibiotic for conjunctivitis, when comparing the average with the lowest category. The odds of receiving an inappropriate antibiotic or benzodiazepine significantly decreased as the size of the GP practice increased. ARMs also showed a similar decrease as the size of the GP practice increased. However, this was only found to be significant in cases of the largest practice sizes.

### Strengths and limitations

A strength of this study is that low-value care among Dutch GPs was assessed for patients receiving an inappropriate prescription using routinely collected, nationally representative data over 4 consecutive years. Furthermore, the use of high-quality and complete clinical data made it possible to distinguish appropriate from inappropriate care.

This study also has some limitations. There is an inherent uncertainty in identifying whether a prescription is of low value. Recommendations contain terms that do not map directly to data variables; also, diagnosis and procedure codes may not precisely identify patients for whom care is of low value. For example, the recommendations regarding conjunctivitis and ARMs were not described with enough detail or required variables that are absent in the data to distinguish appropriate from inappropriate prescribing. The recommendation regarding conjunctivitis requires identifying patients with conjunctivitis caused by a banal pathogen, and who are at high risk. The information required to distinguish the cause of an episode of conjunctivitis is not recorded within the Nivel-PCD and therefore not available in the data used. It also was not possible to identify patients who were at high risk, as the recommendation and the guideline did not provide sufficient detail on the definition of the high-risk population to be able to distinguish them (if the information was available — the information required to identify high-risk patients was often lacking or inadequately described). The current findings therefore could be an overestimate. However, it is not anticipated that these factors will have a major effect on the outcome, as conjunctivitis is most commonly caused by a banal pathogen.^50^

Furthermore, in this analysis of chronic ARM prescriptions, the guideline states that gastro-protection using a non-selective non-steroidal anti-inflammatory drug (NSAID) is justified if a patient is using a high dosage of an NSAID. However, information regarding the dosage of the prescribed NSAIDs was not present within the data used. It therefore was not possible to include these requirements in the current analysis. It is possible that the findings might under-or overestimate the magnitude of the problem.

Also, it was not possible to identify patients with chronic heartburn, as it was only possible to access diagnoses established within the years examined. Patients diagnosed with heartburn outside of this period could therefore not be identified. In addition, heartburn is often only present for a short period of time until ARMs are prescribed. The prescription of ARMs often resolves the patient’s complaints resulting in removal of the diagnosis from the patient’s medical records, making it difficult to define chronic heartburn.

In addition, there was not access to practice and physician characteristics, which could explain variation in prescribing behaviour between GP practices, for example, information on the number of physicians and their age or sex.^51^^–^53

Both the antibiotic and benzodiazepine recommendations are directly linked to specific diagnoses, thereby making their evaluation relatively straightforward. However, this is not the case for the recommendation of chronic ARMs use, which makes its evaluation difficult and more uncertain in comparison with the other recommendations.

Lastly, the final logistic models reported moderate C-statistics. This suggests that these models are unsuitable for predicting, reliably, the risk of patients receiving any of the low-value GP prescriptions. Receiving low-value GP care could have been influenced by other patient or GP characteristics that are not available in the data.^54^

### Comparison with existing literature

Previous studies from Australia, the US, and the Netherlands reported between 60% and 80% of patients with infectious conjunctivitis received a non-indicated antibiotics prescription, which is higher compared with the findings in the current study.^20^^,^^25^^,^^55^ The differences between the findings could be explained by differences in the data sources, study designs, and populations included. For example, Shekhawat *et al* used a patient-indication lens and included all GPs in their analysis,^25^ whereas Cherry *et al* included only patients who visited GP registrars in their sample, resulting in distinct assessment denominators and outcomes.^55^

The current findings relating to low-value prescribing of benzodiazepines for non-specific lower back pain show that Dutch GPs mostly adhere to professional guidelines. Only 3% of patients received an inappropriate prescription, which is lower compared with the findings of recent studies from the US.^56^^,^^57^ Agarwal *et al* reported that 8.5% of patients with back or chronic pain received a benzodiazepine prescription.^56^ Furthermore, Azad *et al* reported that 11.5% of US patients new to opioids with lower back pain received a benzodiazepine within 12 months of their diagnosis.^57^ However, it is difficult to compare these finds with the ones in the current study as patient-population lenses were used in the US studies, whereas this study applied a patient-indication lens resulting in different denominators being used.^15^^,^^42^

No studies looking at non-indicated chronic ARM use were found within the literature by the authors as most studies have focused solely on chronic proton pump inhibitor (PPI) use. However, as only approximately 3.5% of all ARM prescriptions in the current assessment did not concern PPIs, this assessment closely resembles those that solely focus on PPIs. The findings in the current study show that non-indicated chronic use of ARMs is highly prevalent in the Netherlands. However, this is only slightly higher compared with what is reported in the international literature. According to recent literature, between 30% to 80% of PPI prescriptions have no appropriate indication.^32^^–^37^,^^58^ Again, these high levels of variation could be explained by differences in population characteristics, study design — such as the inclusion of all ARMs — and setting. Furthermore, unlike other studies, the current study did not limit the analysis to a specified population, such as older people.^34^^,^^59^ According to Dutch and international guidelines, older adults have more indications justifying the use of a PPI, which might have affected the prevalences reported.

The high levels of inappropriate antibiotic prescriptions could be explained by GPs experiencing patient pressure to provide low-value care. Previous research shows that patient pressure and the GP’s need to maintain a good patient–physician relationship could induce inappropriate prescribing.^60^ Furthermore, the low level of inappropriate benzodiazepine prescriptions could be explained by a long-term policy that promotes cautious prescribing of benzodiazepines because of their addictive properties. Finally, the high prevalence of inappropriate chronic ARM prescriptions could be because of their reputation, at least, for being harmless. ARMs, in contrast to antibiotics and benzodiazepines, are commonly sold over-the-counter at most pharmacies in the Netherlands. The authors anticipate that the findings here are most likely an underestimate, as it was not possible to capture details of all the people who have used ARM for extended periods in this study as non-prescription ARM use is not included in this analysis.

### Implications for research and practice

The findings relating to these three low-value pharmaceutical GP prescriptions demonstrate that both antibiotics for conjunctivitis and the chronic use of ARM are prescribed inappropriately; there is no indication that this will greatly decline over time. This suggests a joint national effort is required to change prescribing behaviour. For such an effort, detailed insight into the views of patients and prescribers, and the barriers and facilitators for the withdrawal of inappropriate medication — de-prescribing — is required to design a tailor-made de-prescribing strategy. Furthermore, the observation that little to no correlation exists between the low-value prescription rates of the three types of low-value pharmaceutical care within practices suggests that the problem of low-value pharmaceutical GP care cannot be addressed through a single de-prescribing strategy. But, rather, it requires de-prescribing strategies that are tailor-made to the type of pharmaceutical care that one aims to address. The assessment methods used in this study could be used to monitor changes resulting from any interventions. Furthermore, the findings relating to potential patient characteristics that are associated with the increased odds of receiving any of the low-value prescriptions examined could provide some focus for policy interventions.

In conclusion, this research shows that low-value pharmaceutical care is prevalent among Dutch GPs, but its prevalence varies depending on the clinical problem. Between 2016 and 2019 many patients received an inappropriate antibiotic or chronic ARM prescription. Benzodiazepines for lower back pain were generally prescribed in line with the guidelines. Among the three types of low-value pharmaceutical care, large variation between GP practices and general variation in prescribing were observed. These insights may help in designing a national campaign to change this behaviour.

## Data

Data are available on request and under conditions specified by Nivel-PCD.
